# Desmoglein 3: A Help or a Hindrance in Cancer Progression?

**DOI:** 10.3390/cancers7010266

**Published:** 2015-01-26

**Authors:** Louise Brown, Hong Wan

**Affiliations:** 1 Queen Mary University of London, Barts and the London School of Medicine and Dentistry, Center for Clinical and Diagnostic Oral Sciences, Institute of Dentistry, Blizard Building, London E1 2AT, UK; E-Mail: louise.brown@liverpool.ac.uk; 2 Department of Cellular and Molecular Physiology, University of Liverpool, Institute of Translational Medicine, Liverpool L69 3BX, UK

**Keywords:** desmoglein 3, cell signalling, desmosomes, cell-cell adhesion, squamous cell carcinoma, cancer biomarker, cancer progression, cancer suppression, metastasis

## Abstract

Desmoglein 3 is one of seven desmosomal cadherins that mediate cell-cell adhesion in desmosomes. Desmosomes are the intercellular junctional complexes that anchor the intermediate filaments of adjacent cells and confer strong cell adhesion thus are essential in the maintenance of tissue architecture and structural integrity. Like adherens junctions, desmosomes function as tumour suppressors and are down regulated in the process of epithelial-mesenchymal transition and in tumour cell invasion and metastasis. However, recently several studies have shown that various desmosomal components, including desmoglein 3, are up-regulated in cancer with increased levels of expression correlating with the clinical stage of malignancy, implicating their potentiality to serve as a diagnostic and prognostic marker. Furthermore, *in vitro* studies have demonstrated that overexpression of desmoglein 3 in cancer cell lines activates several signal pathways that have an impact on cell morphology, adhesion and locomotion. These additional signalling roles of desmoglein 3 may not be associated to its adhesive function in desmosomes but rather function outside of the junctions, acting as a key regulator in the control of actin based cellular processes. This review will discuss recent advances which support the role of desmoglein 3 in cancer progression.

## 1. Introduction

Desmosomal cadherins constitute the adhesive interface at the core domain of desmosome junctions (DSMs), which in principle function to anchor the intermediate filaments of neighbouring cells to sites of adhesion to form the desmosome-intermediate filament complex essential in the maintenance of tissue architecture and integrity [1]. Since cell-cell adhesion is a prerequisite in many cellular processes DSMs have been implicated in epithelial polarisation, proliferation, stratification, differentiation and morphogenesis as well as embryonic development through their ability to influence intercellular signal transduction [1,2]. A characteristic desmosome is essentially composed of three protein families arranged to form two symmetrical electron dense cytoplasmic plaques between adjacent cells that flank a shared central extracellular core domain [3,4]. Desmosomal cadherins, desmogleins (Dsg) 1–4 and desmocollins (Dsc) 1–3 mediate cell-cell adhesion and serve as a scaffold for the assembly of the cytoplasmic dense plaques. Armadillo proteins, plakoglobin (Pg) and plakophilins (Pkp), associate with the cytoplasmic tails of desmosomal cadherins to form the outer dense plaque which in turn associates with the *N*-terminal head domain of the Plakin family protein, desmoplakin (Dp), which links the stress-bearing intermediate filaments of the cytoskeleton to desmosome via its *C*-terminal domain, forming the inner dense plaque [1,5]. Another essential component is a p53 apoptosis effecter related to the PMP-22 (Perp). Although Perp has been proven to be critical in DSM assembly and maintenance its interacting partners currently remain unknown [6,7].

The tumour suppressor function of DSMs is believed to be achieved through mediating strong cell-cell adhesion and sequestering desmosomal components, some of which may have oncogenic potential, to enforce adhesion and to prevent epithelial to mesenchymal transition (EMT) and tumour development [8,9]. Paradoxically, evidence has emerged that shows an upregulation of some desmosomal components, such as Dsg2, Dsg3 and Pkp3 in cancer, in association with cancer progression and/or reduced survival [10,11,12,13,14,15]. There are several reports based on large cohort studies that suggest a pro-cancerous role for Dsg3 with a clear correlation between its expression levels and clinical stage and metastasis [11,15,16,17,18,19]. In line with this, *in vitro* studies based on overexpression of human Dsg3 in cancer cell lines have shed light on its signalling role that regulates a variety of cellular processes including cell cohesion and migratory behaviour. This review will focus on recent advances with regard to the role of Dsg3 in cancer progression and metastasis.

## 2. Desmosomes in Tumour Suppression

Intercellular junctions are conventionally accepted to function as tumour suppressors by restricting cell growth through contact-mediated inhibition of cell proliferation and locomotion [20,21]. Disruption of cell-cell adhesion has been shown to contribute significantly to uncontrolled cell proliferation and plays a causal role in EMT, cancer formation, progression and subsequent tumour dissemination (reviewed by [22]). In the process of EMT, dissolution of intercellular junctions including both DSMs and adherens junctions, in addition to other cellular and molecular events, occurs in epithelial cells that facilitates conversion from benign to metastatic tumours [9,23,24]. The tumour suppressor function of cell-cell adhesion is exemplified by the loss of E-cadherin, a hallmark of EMT, and its down-regulation is directly associated with the loss of contact inhibition and tumour development [25,26,27]. In accord, the expression of E-cadherin in cancer cells inhibits cell growth with a concomitant reduction of cell invasiveness [28,29,30]. Similarly the expression of desmosomal components, Dsc1a and Dsc1b, Dsg1 and Pg has been shown to provoke cell cohesion and inhibit motility and invasion [31,32,33]. Correspondingly, the loss of desmosomal components, Perp, Dsg1-3, Dsc2, Dsc3, Pg, Dp and Pkp1-3 is found in the development and progression of squamous cell carcinoma (SqCC), including that of the head and neck, skin, prostate and bladder [8].

The mechanisms by which DSMs suppress tumour formation are as diverse as the molecules that attribute to their function. Many studies suggest that this may be achieved by regulating the availability of desmosomal components with oncogenic potential, such as Pg and Pkps, to suppress tumour development [8,9]. Plakoglobin, a closely related homologue of β-catenin, is shown to exhibit β-catenin-like activity and modulate Wnt/β-catenin signalling by displacing adherens junction proteins [34,35]. This is believed to be achieved through its translocation to the nucleus, complex formation and activation with LEF/TCF transcription factors [35,36]. Indirectly Pg has also been theorised to modulate Wnt/β-catenin signalling by displace adherens junction-associated β-catenin or blocking its cytoplasmic degradation [9,37] to permit its cytoplasmic accumulation, subsequent nuclear translocation and activation of β-catenin-LEF/TCF target genes [36]. Thus it was reasoned that, by sequestering Pg to the plasma membrane DSMs, like adherens junctions, are able to influence Wnt/β-catenin signalling to suppress tumour development [8,9]. However, it is worth noting that Pg has also been shown in numerous studies to negatively regulate Wnt/β-catenin signalling by inhibiting TCF/LEF transcriptional activity through direct interaction [38,39,40] (discussed below).

The newly characterised desmosomal component Perp has emerged as a crucial mediator in tumour suppression through its function on DSM formation as well as its ability to induce apoptosis [8]. This is achieved through its transcriptional regulation by p63 during epithelial development and morphogenesis to promote cell-cell adhesion and also by the p53 tumour suppressor in response to DNA damage and oncogenic stress to induce apoptosis [6,41]. For more detail regarding DSMs in tumour suppression see [9,41].

### 2.1. Desmoglein 3 in Tumour Suppression

Evidence of the Dsg3 down-regulation in the context of cancer development and progression is comparatively less. However, a reduction of Dsg3 expression has been observed in moderately or poorly differentiated oral SqCC compared with normal oral epithelia [42] and immortalised oral SqCC cell lines compared to mortal oral SqCC and normal oral keratinocytes [43]. Down regulation of Dsg3 is also found to be associated with a loss of differentiation and enhanced metastasis in uterine endometrial and oral squamous cell carcinoma [44,45].

### 2.2. Lessons from Pemphigus Vulgaris

Our understanding of Dsg3 function is largely based on studies in Pemphigus Vulgaris (PV), a rare autoimmune blistering disorder. Characterised by the loss of keratinocyte cohesion in the oral mucosa and epidermis, PV is caused by cell surface binding of auto-antibodies (PV-IgGs) that target mainly the extracellular domain of Dsg3 and trigger a range of cellular and molecular events. Such studies have uncovered the fundamental role of Dsg3 in cell-cell adhesion as well as in signal transduction. The addition of PV-IgGs to keratinocyte culture has been shown to promote rapid re-organisation of cortical actin filaments [46,47] and to induce the phosphorylation of Dsg3 concomitant with its dissociation from DSMs and rapid endocytosis. Other events observed include increased intracellular calcium concentrations and activation of various signalling molecules such as Pg, PKC, p38 MAPK, heat shock protein p27, Src and c-Myc [46,48,49,50,51,52]. Taken together, these findings support the signalling role of Dsg3 which could potentially contribute to cancer development or suppression. Interestingly, an increased incidence of internal malignancy in the PV patients has been reported in the literature [53,54]. While this association may be attributed to the effect of immunosuppressive therapy administered to PV patients, or as an immunological consequence associated with autoimmune disease, it appears plausible that the development of cancer in these patients could be partly attributed to the loss of Dsg3 function. Further study in carcinogenesis using the active PV model may provide information about the link between PV and tumour development.

## 3. Desmoglein 3 in Cancer Progression

Despite the traditional view of DSMs as tumour suppressors and the contribution of Dsg3 in desmosomal adhesive function, recent studies have discovered that Dsg3 is upregulated in SqCC of the head and neck, lung, skin and oesophagus *etc.* [11,15,16,17,18,19]. Furthermore, a correlation between the expression levels of Dsg3 and tumour progression and metastasis has been reported [11,16,55], suggesting that in a given context Dsg3 may contribute to cancer progression. Adopting a gain-of-function approach, overexpression of wild type human Dsg3 in A431 and SqCC/Y1 carcinoma cell lines has been shown to significantly increase cell spreading, membrane protrusion and dynamics as well as cell migration and invasion, a phenotype that could be suppressed by Dsg3 silencing [56,57,58]. These findings are consistent with the reports adopting a loss-of function approach where Dsg3 silencing in oral SqCC cells results in suppression of tumour cell growth, migration and invasion *in vitro* and *in vivo* [11,43,59].

## 4. How Desmoglein 3 Overexpression Promotes Cancer Progression

### 4.1. Regulation of Rho GTPases and the Actin Cytoskeleton

Cell migration is a highly integrated multistep process initiated by actin based membrane morphological changes, specifically membrane protrusions [60]. The driving forces behind the formation and maintenance of pro-invasive and migratory structures require the intricate spatial and temporal regulation of the actin cytoskeleton [61,62]. Not surprisingly, aberrant modulation and organisation of the actin cytoskeleton plays a pivotal role in cancer progression and metastasis [62]. Mechanistic studies of PV have demonstrated that binding of PV-IgGs to cultured keratinocytes provokes cortical actin reorganisation [46,47,52] through Rho GTPase RhoA and to a lesser extent Rac1 and Cdc42 [46,47,50,51,52]. In the context of cancer development and progression, Rho GTPases are known to play a critical role in processes including tumourigenesis, cell-cycle control, invasion and metastasis [63]. The overexpression of Dsg3 in carcinoma cells has been shown to increase Rac1 and Cdc42 activities and to a lesser extent RhoA, accompanied by pronounced lamellipodia and filopodia and an enhanced rate of actin turnover [58]. These findings support the regulatory activity of Dsg3 in actin organisation and dynamics through the mechanism involved in Rho GTPases in cancer cell biology.

### 4.2. Organisation of Specialised Membrane Domains

The special regulation of actin based processes requires orchestration in order to achieve a global cellular response [64]. Ezrin, Radixin and Moesin or ERM family proteins are identified to play a key role in these processes through their cross-linking activity and recruitment of multi-protein complex to specific sub-cellular compartments [65]. Enriched in cell-surface structures, such as microvilli, filopodia, uropods and membrane ruffles [66,67,68,69], the ERM proteins have been shown to participate in the formation and maintenance of these structures to modulate cellular processes including polarity, adhesion, spreading and motility [70,71,72,73,74,75,76]. Furthermore, the ERM proteins are also found to be dynamically regulated during cancer progression, with Ezrin being upregulated during early metastatic progression and expansion but down regulated during establishment and survival in metastatic nodes [77,78]. Altered expression, phosphorylation and sub-cellular localisation of Ezrin is also correlating with enhanced migration, invasion and metastasis in a variety of cancers [79,80,81,82,83,84,85,86]. The potential of Ezrin as a downstream effecter of Dsg3 has recently been explored suggesting it plays a part in facilitating Dsg3-dependent regulation of pro-migratory and invasive structure in a malignant setting [56]. In this report, the overexpression of Dsg3 enhanced phosphorylation and localisation of Ezrin at basal plasma membrane domains [56,87], events indicative of the aberrant regulation of Ezrin in cancer progression [88].

### 4.3. Regulation of Src Signalling

The Src family of non-receptor tyrosine kinases, of which Src, Yes and Fyn are ubiquitously expressed, is a group of enzymes that propagate a diverse spectrum of receptor-induced biological activities through their ability to engage with different classes of cellular receptors and numerous cellular targets [89]. Activated following ligand-receptor engagement, Src is integral to the maintenance of normal cell homeostasis through the regulation of various functions including proliferation, adhesion, differentiation, gene transcription, cytoskeletal alterations and migration [89,90,91]. Not surprisingly, the aberrant modulation of Src activity is associated with abnormalities in specific cell types, tissues and physiological responses [89]. Moreover, the overexpression and/or hyper-activity of Src is associated with a variety of carcinomas, including colorectal, breast, head and neck and lung [90,91,92,93]. While its transforming ability is unlikely [94], the role of Src in tumour progression and metastasis is well established with increased Src activity being associated with hyper-proliferation, EMT, migration, invasion and metastasis [91,95,96]. The proposed mechanisms of the Src activation in a malignant setting are well documented [89,96]. As activating mutations and genomic amplifications in Src are rare, aberrant activation of Src is thought to be accomplished through upstream receptors (direct or indirect) and alteration in upstream kinases such as Csk and phosphatases that influence the intermolecular interactions, localisation and net phospho-status of Src [89,96]. Recent studies suggest that Dsg3 is able to act as a cell surface regulator in Src activation and this signalling pathway seems to be involved in E-cadherin assembly and adhesion. Increased expression of Dsg3 in cancer cell lines has been demonstrated to elicit a significant increase in the tyrosine phosphorylation of Src and its downstream targets of adherens junction proteins, E-cadherin, β-catenin and p120 with a consequence of decreased E-cadherin expression [57,97,98,99]. Src-mediated tyrosine phosphorylation of E-cadherin leads to its ubiquitination and lysosomal degradation [100]. The tyrosine phosphorylation of p120 has been reported to increases its affinity for Rho GTPases to potentially promote cell motility [101,102]. The tyrosine phosphorylation of β-catenin enhances the LEF/TCF transcriptional activity through Wnt/β-catenin signalling pathway.

Although it has been demonstrated that Dsg3 is capable of regulating Src activity it remains unclear how this could be achieved. Emerging evidence suggests that this regulatory pathway may involve caveolin-1, a major constituent of caveolae. Caveolae are a special type of lipid raft of the plasma membrane with a flask-like structure which functions as a regulator in endocytosis and exocytotic vesicle trafficking, cell adhesion and motility [103,104]. It is thought that caveolae modulate signal transduction through the compartmentalisation of specific signalling molecules and the regulation of their activity [105,106,107]. Caveolin-1 is shown to negatively regulate Src activation through an inhibitory interaction which prevents its auto-phosphorylation [106,107]. Amino acid sequence analysis of the Dsg subfamily identified a sequence in Dsg3 that potentially binds the scaffolding domain of caveolin-1 [108]. In support, the same region in Dsg2 has been demonstrated to bind directly to caveolin-1 [109]. It is likely that Dsg3 regulates Src activation by competing with inactive Src for a binding site on the scaffolding domain of caveolin-1 that in turn leads to release of Src followed by its auto-activation. Furthermore, the close association between Dsg3 and caveolin-1 has been demonstrated biochemically and microscopically by several independent studies [108,109,110].

### 4.4. Regulation of Wnt/β-catenin Signalling

In attempt to elucidate the molecular mechanism of Dsg3-dependent tumour cell migration a recent study by Chen *et al.* suggests that Dsg3 negatively regulates Wnt/β-catenin signalling in a Pg-dependent manor, most likely by sequestering Pg preventing its nuclear translocation and suppression of LEF/TCF transcriptional activity [59]. In support, other independent studies provide evidence suggesting an inhibitory role for Pg in Wnt/β-catenin/TCF pathway [38,39]. LEF/TCF transcription factors are activated through the Wnt signalling pathway which plays an essential role in development, cell proliferation, survival and migration. Aberrant signalling of Wnt/β-catenin/TCF pathway has been shown to result in defects in embryonic development and a wide range of adult pathologies, most prominently cancer [111,112,113,114]. The study by Chen *et al.* showed that Dsg3 silencing in head and neck SqCC increased the translocation of Pg to the nucleus where it interacts with and inhibits TCF/LEF transcription activity, to suppress tumour growth and invasion [59]. Correspondingly, increased Dsg3 levels were shown to be correlated with reduced nuclear Pg accompanied with elevated expression of the LEF/TCF transcriptional targets, cyclin D1, c-Myc and MMP7 in both the tissues of head and neck cancer patients and oral SqCC cell lines [59]. Thus, the upregulation of Dsg3 in oral SqCC could potentially tip the balance in the favour of the β-catenin-LEF/TCF interaction and activation. In support, the increased β-catenin expression, nuclear localisation and activity in oral SqCC has been reported in association with increased Wnt signalling [115]. In line with this notion, an *in vitro* study based on cancer cell lines shows that Dsg3 overexpression increases the tyrosine phosphorylation and activity of β-catenin as well as a reduction of E-cadherin [57,116]. Both the reduction of E-cadherin and tyrosine phosphorylation of β-catenin could promote the cytoplasmic accumulation of β-catenin and its subsequent translocation to the nucleus with a consequence of LEF/TCF transcription activation [117].

### 4.5. Regulation of the Transcription Factor, Activator Protein-1

Activator protein-1 (AP-1) is a dimeric transcription factor with an expansive transcriptional repertoire propagated by its diverse homo- and hetero-dimeric compositional array of Jun, Atf, Fos and Maf bZIP protein families. The activity of AP-1 is regulated at multiple levels; transcriptionally the abundance of AP-1 components can be influenced by various extracellular signals transduced through MAPK cascade. For example, the activation of JNK in turn activates ternary complex factors (TCFs) to induce the cFos expression [118] whereas the activated p38 induces the cJun expression [119]. Post-transcriptionally, the activity of both pre-existing and newly synthesized AP-1 components can be achieved through the protein phosphorylation. With respect to cJun, PKC-dependent phosphorylation of the *N*-terminal residues inhibits DNA binding of cJun homodimers [120], while the phosphorylation of cJun at serine 73 and to a lesser exstent serine 63, by JNK enhances its ability to activate transcription [121].

AP-1 is known to regulate a vast array of cellular processes including development, proliferation, apoptosis, immune and stress responses [122,123,124,125]. The deregulation of AP-1 has been associated with a variety of pathologies including inflammatory disease of the bone, skin and liver [126,127] and upregulated in numerous malignancies, including skin, breast, cervix and lung [87,128,129,130,131] through its ability to regulate genes associated with EMT and the migration-invasion programme [24,132,133,134,135,136,137]. Recently, it has been shown that Dsg3 overexpression enhances cJun phosphorylation at serine residues 63 and 73 [56,138] which translated into enhanced c-Jun:AP-1 transcriptional activity that could be abrogated by Dsg3, JNK, PKC, p38 and Src inhibition [56,138]. Furthermore, this MAPK-dependent mechanism appears to be universal as it can be demonstrated in various cell types, including Cos-1 fibroblastic-like cells [138]. In support, other study showed that cJun interacts with TCF4 to form a tertiary complex, containing c-Jun, TCF4 and β-catenin, that binds to cJun promoter thus conferring transcription activity, in a JNK-dependent manner, in intestinal tumourigenesis [139].

### 4.6. Role of Desmoglein 3 in Carcinogenesis

Although Dsg3 is shown to play a positive role in oncogenesis evidence suggests that Dsg3 may not function as a main driver in cancer formation but rather as a factor that promotes cancer progression. The miss-expression of Dsg3 in the epidermis of transgenic mice driven by the K1 promoter provokes hyper-proliferation, abnormal differentiation and parakeratosis of the epidermis [140]. While these characteristics are also observed in diseases associated with increased cell turnover including SqCC, no evidence of tumour development was reported in this study [140]. Using two models of skin carcinogenesis a recent study investigated the role of Dsg3 in cancer suppression [141]. In the first approach the tumourigenic potential of transformed Dsg3^−/−^ and Dsg3^+/−^ keratinocytes was examined in immune-compromised *scid* mice. A reduction in tumour formation and growth was observed in mice injected with Dsg3^−/−^ keratinocytes compared with control group injected with Dsg3^+/−^ keratinocytes [141]. In the second approach UVB-induced SqCC formation was analysed in *Dsg3*^−*/*−^ mice alongside their wild type counterpart. This approach revealed no significant difference in tumour latency, size and multiplicity between the two cohorts [141]. Collectively, these studies suggest that the ablation of Dsg3 does not appear to promote tumour development, implying Dsg3 does not possess tumour suppressive ability. Taken together, these studies are consistent with the notion that Dsg3 plays a positive role in cancer progression and metastasis [11,18,42,55,142].

### 4.7. The Diagnostic, Prognostic and Therapeutic Potential of Desmoglein 3

With more than 90% of cancer related deaths attributed to metastasis rather than the primary tumour [143], the importance of tackling this aspect of cancer cell behaviour is evident. Squamous cell carcinoma represent the most common cancer capable of metastasis [144]. Despite advances in its diagnosis and treatment [145], the four most common SqCCs, non-melanoma skin cancer, head and neck, oesophageal and non-small cell lung cancer [145], carry among the lowest 5-year survival rates (with the exception of non-melanoma skin cancer) which have not seen significant increases in 30 years [146]. This is in part believed to be attributed to late diagnosis and lack of biological markers which are essential for diagnosis, prognosis and monitoring tumour response to therapy. As the upregulation of Dsg3 has been identified in SqCCs of the head and neck, oesophagus and lung [11,15,16,18,147,148] its potential as a diagnostic and prognostic marker has been explored.

With respect to head and neck SqCC regional lymph node metastasis is common and represents the strongest prognostic factor pertinent to disease staging and treatment strategy selection [149,150,151,152]. With patient survival correlating with the level of nodal involvement [153] and the inherent oversight of micrometastases during clinical staging protocols [154,155], elective nodal dissection (END) followed by pathological examination are carried out as standard procedure. END significantly improves regional recurrence-free survival and lowers the incidences of distal metastasis [156,157,158,159]. Even so, 7%–15% of node-negative patients suffer from recurrence and 50%–70% of patients are over-treated [157,158,160,161,162,163,164]. Sentinel node biopsy presents a feasible and accurate means of avoiding unnecessary END by identifying node negative patients. However, its potential and application is limited by the lack of rapid and accurate methods and markers in the detection of metastatic nodes. Dsg3 has emerged as an accurate biomarker for the detection of lymph node metastasis in head and neck SqCC, discriminating between positive and benign nodes with ~100% accuracy [55,164,165,166]. Furthermore, by adopting the analyses of the Dsg3 expression in sentinel node biopsy using qRT-PCR and microfluidic immunoarray platforms the identification of positive and negative nodes can be achieved within an intraoperative timeframe [55,165,167]. These advances imply that Dsg3 could potentially be used as a maker for clinicians to reduce unnecessary lymph node dissection [168,169] and the frequency of false-negatives to improve diagnostic accuracy and to allow tailoring of treatment strategies for patients with head and neck SqCC.

In addition to head and neck SqCC, Dsg3 has also been shown to be of value in the diagnosis of pulmonary SqCC. Treatment selection for patients diagnosed with lung cancer is predominantly guided by separating lung carcinomas into small cell and non-small cell lung cancers (includes squamous cell carcinoma, adenocarcinoma and large cell carcinoma) and the stage of diseases [170,171,172]. Non-small lung cancers represent a heterogeneous group of cancers that most likely attribute to how patients respond to therapy [173,174]. The current combination of markers including cytokeratin 5/6 and p63 exhibits relatively low sensitivity and/or specificity [18]. Dsg3 has been shown not only to be highly expressed in pulmonary SqCC [17,18,175] but also appears to be a useful ancillary marker to separate pulmonary SqCC from other non-small cell lung cancers [18].

Although Dsg3 has been proposed as a potential therapeutic target in oral SqCC currently limited studies has been reported in the literature. Nevertheless, it was demonstrated by a group of researchers that the RNAi mediated Dsg3 silencing in head and neck SqCC lines reduces tumour growth and metastasis in xenograft studies in BALB/C nude mice [11,59] indicating the therapeutic potential of targeting Dsg3 in preventing cancer progression.

## 5. Future Perspectives

Although a large body of study regarding Dsg3/PVA has been documented in the literature in pemphigus research highlighting its critical role in cell cohesion, recent advances in the last decade have discovered Dsg3 as a potential oncogene and a positive biomarker of SqCC. There is no doubt that Dsg3 functions as a cell-cell adhesion receptor in DSMs. However, accumulating data suggest that Dsg3 also acts as an important surface regulator for various signal pathways that are involved in actin based cell adhesion, morphological change and locomotion. Like Src and some other signalling molecules, Dsg3 seems to act as a pleiotropic gene having influence on both cell-cell adhesion and cell locomotion depending on the context in study. In normal epithelial cells Dsg3 may function to facilitate cell-cell adhesion but in transformed cells where DSM function is impaired, the overexpression of Dsg3 may favour its oncogenic signalling activity that causes pronounced membrane protrusions and accelerated cell locomotion. However, there are still many questions unanswered. For example, it remains unclear the precise function of Dsg3 in tumour cell biology and whether it acts as a driver in tumour formation? What mechanism regulates its gene expression in normal and cancer cells? Are there any other environmental factors/cues in addition to calcium? Does the overexpression of Dsg3 in cancer involve post-translational regulation, like E-cadherin? As for the Dsg3 signalling, it remains unclear what triggers its activation? Does Dsg3 signalling occur on the cell surface and also in the cytoplasm and if it is on the surface, is it triggered by homophilic ligation, such as trans- and/or cis-binding? It might well be that certain transitional structural conformation of Dsg3 or protein dimerisation on the surface is required for its signalling action and when DSMs are established this structural conformation has undergone a different rearrangement. While this review outlines the potential participation of Dsg3 in cancer cell migration and invasion in a non-adhesive fashion, this by no means negates its role of cell-cell adhesion in collective cell migration and invasion which is an important aspect of the metastatic process [176,177]. Nevertheless, the consistent findings in SqCC have placed Dsg3 as a promising biomarker in cancer and a potential therapeutic target in preventing cancer progression and metastasis. The clarification of this additional function for Dsg3 will advance our understanding the role of Dsg3 in tumour cell biology and may have implication in the development of anti-cancer therapy.

## 6. Conclusions

It is well established that Dsg3 is targeted by pemphigus autoantibodies and plays a crucial role in pemphigus pathogenesis. Recent studies have shed light that Dsg3 also acts as a key regulator in various intracellular signal pathways that are likely hijacked in cancer and promote cancer cell invasion and dissemination. However, our understanding on the functions of this gene in tumour cell biology remains limited. Further study of Dsg3 in cancer cells and their microenvironment is required to advance our knowledge. Furthermore, *in vivo* investigation in transgenic model with Dsg3 overexpression will particularly benefit by providing new insights into how exactly this gene is involved in cancer development and progression.
